# The pleiotropic nature of NONO, a master regulator of essential biological pathways in cancers

**DOI:** 10.1038/s41417-024-00763-x

**Published:** 2024-03-16

**Authors:** Domenica Ronchetti, Valentina Traini, Ilaria Silvestris, Giuseppina Fabbiano, Francesco Passamonti, Niccolò Bolli, Elisa Taiana

**Affiliations:** 1 Department of Oncology and Hemato-Oncology, University of Milan, Milan, Italy; 2 Hematology, Fondazione IRCCS Cà Granda Ospedale Maggiore Policlinico, Milan, Italy

**Keywords:** Cell biology, Cancer genetics

## Abstract

NONO is a member of the Drosophila behavior/human splicing (DBHS) family of proteins. NONO is a multifunctional protein that acts as a “molecular scaffold” to carry out versatile biological activities in many aspects of gene regulation, cell proliferation, apoptosis, migration, DNA damage repair, and maintaining cellular circadian rhythm coupled to the cell cycle. Besides these physiological activities, emerging evidence strongly indicates that NONO-altered expression levels promote tumorigenesis. In addition, NONO can undergo various post-transcriptional or post-translational modifications, including alternative splicing, phosphorylation, methylation, and acetylation, whose impact on cancer remains largely to be elucidated. Overall, altered NONO expression and/or activities are a common feature in cancer. This review provides an integrated scenario of the current understanding of the molecular mechanisms and the biological processes affected by NONO in different tumor contexts, suggesting that a better elucidation of the pleiotropic functions of NONO in physiology and tumorigenesis will make it a potential therapeutic target in cancer. In this respect, due to the complex landscape of NONO activities and interactions, we highlight caveats that must be considered during experimental planning and data interpretation of NONO studies.

## Introduction

NONO (NONO/p54nrb, or non-POU domain-containing octamer-binding protein) is a member of the Drosophila behavior/human splicing (DBHS) family of proteins, together with the splicing factor proline-/glutamine-rich (SFPQ) and the paraspeckle protein component 1 (PSPC1) [1].

NONO is a multifunctional protein with numerous roles in genome maintenance and gene regulation [1, 2]. It is mainly located within the nucleus of most mammalian cells; however, under different conditions, it can be triggered by binding to local high concentrations of various nucleic acids to form microscopically visible nuclear bodies, paraspeckles (PSs) or large complexes such as DNA repair foci [1]. In particular, NONO is an essential structural component of PSs, which are dynamic organelles that regulate different cellular functions, including mRNA nuclear retention, micro-RNA processing, DNA damage repair (DDR) systems regulation, stress response, and act as a molecular sponge for RNA binding proteins [3].

The NONO protein has two N-terminal RNA recognition motifs (RRM), a NONA/paraspeckle domain (NOPS), a coiled-coil at the C-terminus, and N- and C-terminal intrinsically disordered low complexity domains [1, 4]. Structural and biological data regarding NONO suggest that it rarely operates as a single molecule; rather it forms heterodimers with the other DBHS proteins, which can bind DNA and RNA, and mediates additional protein–protein interactions [4, 5]. Furthermore, NONO undergoes liquid–liquid phase separation, a process in which macromolecules such as proteins or nucleic acids condense into a dense phase that often resembles liquid droplets, and this dense phase coexists with the dilute phase; this phenomenon explains the dynamic association of molecules into membrane-less organelles, or condensates [6–9]. Indeed, both PSs and DNA damage foci are now classified as condensates built upon liquid–liquid phase separation properties of various protein components, including NONO [9–11]. Recently, it has been reported that phase separation promoted by NONO can play an important role also in gene regulation [12].

Several studies have identified DNA and RNA substrates bound by NONO in different biological contexts [13–16]. These studies have revealed widespread binding to diverse gene regulatory elements in chromatin as well as binding to mainly intronic elements of pre-mRNAs. Overall, these different interactions suggest that NONO protein acts as a “molecular scaffold” to carry out versatile activities in many aspects of gene regulation. Indeed, NONO engages in almost every step of gene regulation [4], including mRNA splicing [17–20], activation of transcription [19, 21], termination of transcription [22], DNA unwinding [4, 23], and nuclear retention of defective RNA [24, 25].

There is a large amount of data showing that NONO exerts its various functions through multiple mechanisms, and participates in many biological processes including cell proliferation, apoptosis, migration, and DDR [1–3, 26, 27]. Its fundamental role in maintaining cellular circadian rhythm coupled to the cell cycle has also been demonstrated [28–30].

Besides these physiological activities, emerging evidence strongly indicates several roles for NONO in tumorigenesis. To date, all DBHS proteins have been found to be associated with cancers as either oncogenes or tumor suppressors [1, 2]. NONO is mostly overexpressed in various types of cancers, including lung cancer [31, 32], prostate cancer (PCa) [33, 34], multiple myeloma (MM) [35], melanoma [36], malignant pleural mesothelioma [37], breast cancer (BC) [38, 39], neuroblastoma (NBL) [40], esophageal squamous cell carcinoma [41], and colorectal cancer (CRC) [42]. Increased NONO abundance correlates with the malignant progression of melanoma [36] and breast tumors [38]; furthermore, NONO expression level is an independent prognostic factor for some cancer types [35, 40, 43–45]. In addition to altered expression levels, NONO can undergo various post-transcriptional or post-translational modifications, including alternative splicing (AS) [38], phosphorylation [46], methylation [47], and acetylation [48], whose impact in cancer remains largely to be elucidated. Current knowledge has described that methylation of NONO at R251 by protein arginine methyltransferase 1 (PRMT1) enhances CRC growth and metastasis [42]. Furthermore, in BC, the interaction of the peptidyl–prolyl cis–trans isomerase NIMA-interacting 1 (PIN1) with the C-terminal threonine–proline motifs of NONO increases the stability of NONO by inhibiting its proteasomal degradation, ultimately raising NONO expression levels and activating NONO-induced downstream signaling pathways involved in carcinogenesis [49]. Finally, NONO alterations at the gene level have been found in a subset of papillary renal cell carcinoma. In this case, NONO is involved in the X chromosome inversion inv(X) (p11.2; q12), which results in the fusion of *NONO* gene with the DNA region encoding for the transcription factor E3 (*TFE3*), leading to the production of the TFE3–NONO fusion protein [50]. However, since the activity of this chimeric protein may be independent of NONO, the review will not delve into this topic.

Overall, altered NONO expression and/or function are a common feature in cancer. In the next paragraphs, we summarize the current understanding of the molecular mechanisms and the biological processes affected by NONO in different tumor contexts.

## Breast cancer

Breast cancer (BC) is still the most common cause of cancer death among women worldwide, despite the reduction in mortality due to earlier diagnosis and treatment [51].

BC tissue displays significantly higher NONO expression levels than adjacent normal breast tissue; furthermore, as stated above, NONO abundance and stability are increased by its binding to PIN1, whose expression is also significantly increased in tumor cells [49]. NONO is expressed in BC cells at different levels in estrogen receptor-positive (ER+) and estrogen receptor-negative (ER−) subtypes, based on post-transcriptional regulation. In particular, ER− BC cells generally show a reduced NONO expression with respect to the ER+ subtype. Moreover, a subset of ER+ tumors expresses an amino-terminal truncated isoform of NONO whose biological and molecular activity still remains to be clarified [38].

Regardless of their hormone dependency, recent studies indicate that NONO plays a critical role in the pathophysiology of BC; indeed, NONO functions as an oncogene and affects key cellular processes such as cell proliferation, cell survival, migration and invasion, and stem cell formation [49].

**Fig. 1:**
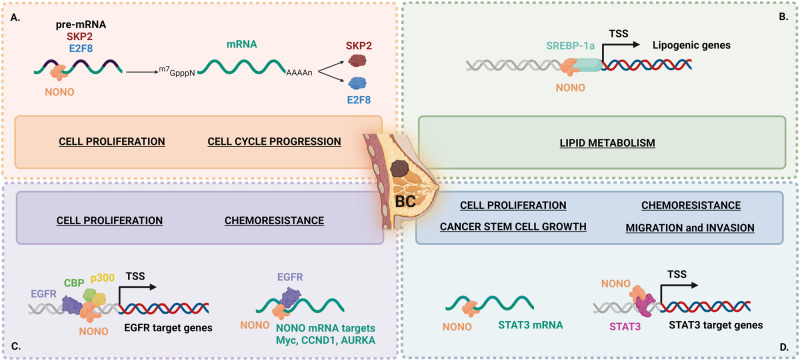
Schematic representation of four different molecular mechanisms altered by NONO deregulation in breast cancer (BC). Simplified pictures of altered mechanisms are shown in (**A**–**D**), see text for detailed description. TSS transcription start site.

NONO contributes to tumorigenesis through different molecular mechanisms (Fig. 1). Specifically, NONO post-transcriptionally regulates the expression of cell proliferation-related genes by binding to their pre-mRNAs and enhancing their splicing. This is the case of both the S-phase-associated kinase 2 (SKP2), an E3 ubiquitin ligase that promotes the degradation of tumor suppressor p27, and E2F transcription factor 8 (E2F8), known to regulate CCNE2 expression [44] (Fig. 1A). Besides its role in RNA processing, NONO can directly bind to certain transcription factors to act as a transcriptional cofactor in regulating gene expression. In detail, NONO binds nuclear sterol regulatory element-binding protein 1a (SREBP-1a) causing an increase of nuclear SREBP-1a protein stability. As a result, NONO stimulates SREBP-1-mediated transcription of lipogenic genes and lipid production, finally contributing to SREBP-1a-dependent BC cells proliferation and tumor growth (Fig. 1B). Interestingly, knockdown of other DBHS proteins, SFPQ, and PSPC1, had no significant effects on the growth of BC cells in vitro, suggesting a NONO leading role in this co-activation activity [39].

Recent data highlighted a peculiar role of NONO in the context of triple-negative BC (TNBC), i.e., tumors that are negative for the expression of the key molecular markers ER, progesterone receptor, and epidermal growth factor receptor, HER2. The TNBC accounts for approximately 15% of all diagnosed BC and is classified as invasive mammary carcinoma with a high rate of recurrence and poor overall survival. In this subset of patients, NONO is highly expressed and closely associated with the malignancy of BC [52]. Specifically, NONO enhances the transcription activity of nuclear EGFR by stabilizing its protein and recruiting the transcriptional co-activator CBP/p300; furthermore, nuclear EGFR enhances the affinity of NONO to its mRNA targets involved in cell proliferation, including Myc, CCND1, and AURKA, ultimately improving their stability [52] (Fig. 1C). It has been also reported that NONO contributes to cancer cell growth and confers drug resistance in TNBC by regulating the signal transducer and activator transcription 3 (STAT3). Indeed, NONO was found both to stabilize STAT3 mRNA and also to directly interact with STAT3 protein increasing its stability and transcriptional activity, thus contributing to its oncogenic function [53] (Fig. 1D).

## Bladder cancer

Bladder cancer (BCa) is the most common genitourinary malignancy [54]; in particular, BCa patients with lymph node (LN) metastasis have an extremely poor prognosis and no effective treatment [55]. NONO is significantly downregulated in LN-metastatic BCa and patients with low NONO expression levels display poorer prognosis [56]. Functionally, NONO markedly inhibits bladder cancer cell migration and invasion in vitro and LN metastasis in vivo. Deregulated NONO expression levels have a significant impact on tumorigenesis by affecting AS, a mechanism that NONO unbalances also in other tumor contexts (see below). AS is a widespread process that leads to structural transcript variations and contributes to proteome complexity, which could result in the expression of proteins with entirely divergent functions. The abnormal regulation of AS is usually coupled with the occurrence and development of tumors. AS is carried out by trans-acting splicing factors, whose deregulation plays a significant role in cell malignant transformation through modulation of the expression of the oncogenic variant [57].

**Fig. 2:**
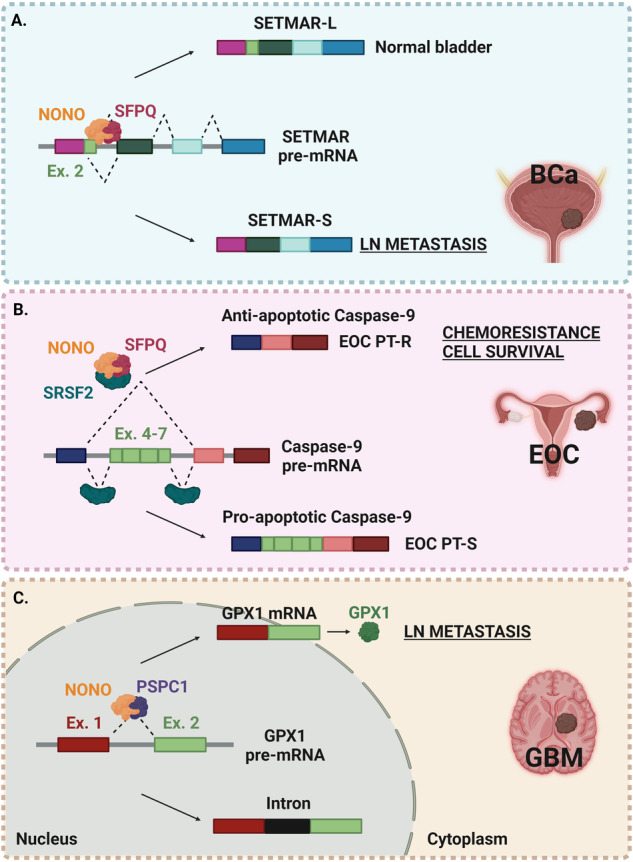
Schematic representation of NONO molecular mechanisms altered by NONO deregulation. **A** Bladder cancer (BCa); **B** Epithelial ovarian cancer (EOC); PT-R and PT-S: resistant or sensitive to platinum, respectively; **C** Glioblastoma (GBM). TSS transcription start site. See the text for a detailed description of the panels.

Specifically in BCa, NONO regulates the exon 2 skipping of the SET and mariner transposase domains-containing protein (SETMAR), a histone methylase with a broad effect on gene expression. In detail, NONO-mediated SETMAR-L expression increases histone methylation, ultimately leading to the transcriptional repression of multiple oncogenes, such as Peroxiredoxin 4 (PRDX4), Glucosidase alpha neutral AB (GANAB), and SET domain-containing protein 7 (SETD7), reported to be associated with cancer metastasis [58–60]. Conversely, NONO downregulation in BCa unbalances SETMAR expression towards the AS transcript variant SETMAR-S, thus losing the inhibitory effect on LN metastasis via SETMAR-L-mediated H3K27me3 on the target genes (Fig. 2A) [56].

## Epithelial ovarian cancer

Epithelial ovarian cancer (EOC) is a relatively rare but highly lethal disease, and its response to platinum (PT)-based chemotherapy directs subsequent treatments and predicts patients’ prognosis [61]. The deregulation of NONO expression in EOC remains to be clarified [62]; however, the literature highlighted an important role for NONO in AS also in this tumor context. It has been demonstrated that NONO and SFPQ are critically involved in EOC cell sensitivity to PT treatment by regulating caspase-9 splicing. Indeed, NONO/SFPQ complex binds to the splicing factor protein SRSF2 which is responsible for caspase-9 mRNA maturation. The relative expression levels of NONO/SFPQ/SRSF2 complex are critical to determine whether exons 4–7 of caspase-9 are retained or excluded from the mature mRNA, leading to different ratios of anti-apoptotic (exons 4–7 skipping) or pro-apoptotic variants of caspase-9 expression. In this manner the NONO/SFPQ complex can protect EOC cells from PT-induced death, eventually contributing to chemoresistance (Fig. 2B). Indeed, the downregulation of NONO/SFPQ complex, combined with PT treatment, can increase the ability of SRSF2 to bind caspase-9 mRNA, thus enhancing the expression of its pro-apoptotic form and consequently cell death [62].

## Glioblastoma multiforme

Glioblastoma multiforme (GBM) is the most common and aggressive primary brain tumor in adults [63]. NONO expression levels are increased in GBM samples in association with poor survival of patients [64, 65]. Functionally, in GBM NONO enhances tumorigenesis by unbalancing AS mechanisms, finally leading to tumor progression and metastasis formation [63]. Specifically, NONO binds to a consensus motif in the intron of the Glutathione peroxidase 1 (GPX1) pre-mRNA in association with another DBHS protein family member, PSPC1. NONO overexpression in GBM unbalances GPX1 splicing towards the productive isoform of the GPX1 enzyme, which protects mammalian cells from oxidative stress [64] (Fig. 2C). In GBM, besides its role in mRNA splicing, NONO affects the Hippo pathway effector TAZ, that promotes cellular growth, survival, and stemness by regulating gene transcription. In particular, NONO enables TAZ liquid–liquid phase separation, which compartmentalizes key cofactors that drive the oncogenic transcriptional program [65].

## Lung cancer

**Fig. 3:**
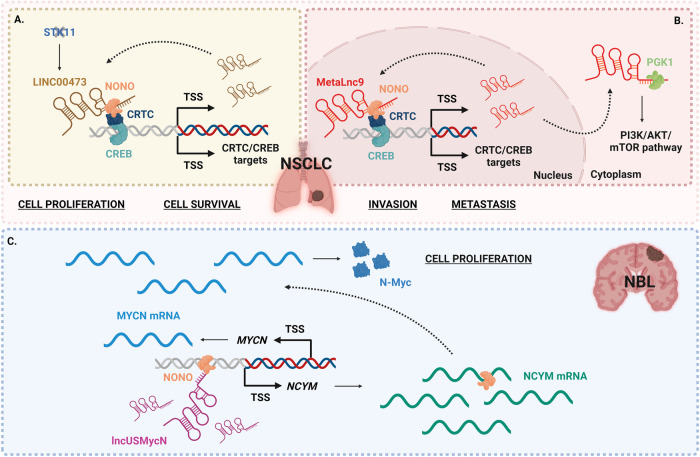
Schematic representation of NONO molecular mechanisms altered by NONO deregulation. Non-small cell lung carcinoma (NSCLC) (**A**, **B**) and Neuroblastoma (NBL) (**C**). TSS transcription start site. See text for detailed description of the panels.

Lung cancer is the leading cause of cancer-related deaths [54]; approximately 80% of lung cancer cases are non-small cell lung carcinoma (NSCLC). NONO is highly expressed in NSCLC tissues as compared with normal ones, and its expression has been correlated with the prognosis of lung cancer [66]. Current data reported that in NSCLC, NONO interacts with long non-coding RNAs (lncRNAs), which are non-protein coding RNAs longer than 200 nucleotides. LncRNAs represent more than half of the mammalian non-coding transcriptome and are involved in many biological processes, such as cell proliferation, apoptosis, and cellular differentiation [67]; moreover, lncRNAs participate in carcinogenesis and tumor progression in many cancer types. In NSCLC, NONO binds two lncRNAs, resulting in an interaction that leads to tumor growth and metastasis. The serine/threonine protein kinase 11 (*STK11*) tumor suppressor gene is mutated in approximately 30% of NSCLC cases, making it the third most common site of genetic alterations in lung cancer after *TP53* and *KRAS* [68]. *STK11* plays critical roles in cell growth, cell polarity, and metabolism. *STK11* inactivation leads to LINC00473 overexpression, which facilitates the recruitment of NONO to CREB-regulated transcription coactivator (CRTC), ultimately promoting the cAMP-mediated transcription of various genes; in addition, LINC00473 acts as a coactivator with CRTC/CREB in a positive feedback mechanism to maintain its own high steady-state levels (Fig. 3A) [31]. Another lncRNA, MetaLnc9, is overexpressed in NSCLC, subsequently causing poor prognosis and enhanced metastasis formation in patients. Like LINC00473, MetaLnc9 interacts with NONO to promote the CRTC-mediated transcription of CREB target genes, including its own expression, thus auto-sustaining its gene expression regulating activity. Furthermore, MetaLnc9 directly binds phosphoglycerate kinase 1 (PGK1) and inhibits its ubiquitin-mediated degradation, leading to the activation of the AKT/mTOR signaling pathway in NSCLC cells (Fig. 3B) [32].

## Neuroblastoma

Neuroblastoma (NBL) is the most common solid tumor in early childhood [63], and high NONO expression levels are associated with poor patient outcome [40]. Approximately 30% of patients with NBL show the amplification of a 130 kb genomic DNA region containing the *MYCN* oncogene, the *MYCN* antisense *NCYM* gene, and the lncRNA lncUSMycN, in association with poor prognosis [69]. N-Myc plays an important roles in cell proliferation and differentiation during embryonic development and induces NBL initiation and progression by regulating target genes expression. In detail, patients carrying this DNA amplification overexpress lncUSMycN that, in association with NONO, upregulates *NCYM* transcription; NCYM mRNA upregulates N-Myc expression by binding to NONO (Fig. 3C) [40, 70]. Despite the knowledge of NONO interaction with this lncRNA, the understanding of the mechanisms of NONO-dependent oncogenic activity in the disease is still poor. A very recent study highlighted a complex NONO-dependent regulation of gene expression, based on the capability of NONO to form RNA- and DNA-tethered condensates throughout the nucleus and undergo liquid–liquid phase separation; in particular, NONO regulates super-enhancer-associated genes, including the transcription factors HAND2 and GATA2 [12], demonstrated to modulate differentiation and migration in NBL [71, 72].

## Colorectal cancer

Colorectal cancer (CRC) is the third most commonly diagnosed cancer worldwide [54]. Clinically, a considerable number of CRC patients develop metastases, such as distant metastasis and LN metastasis. NONO is overexpressed in CRC tissue where it represents a potential biomarker of poor prognosis [42]; moreover, NONO methylation promotes CRC growth and metastasis [42]. It has been reported that CRC metastasis also correlates with the expression levels of the gastric adenocarcinoma predictive long intergenic noncoding (GAPLINC), an lncRNAs that is deregulated in different cancer types [73, 74]. GAPLINC is up-regulated in CRC tissues, and it is involved in the migration and invasion of CRC cancer cells through its binding with NONO and SFPQ [75], based on mechanisms that warrant further investigations.

## Gastric cancer

**Fig. 4:**
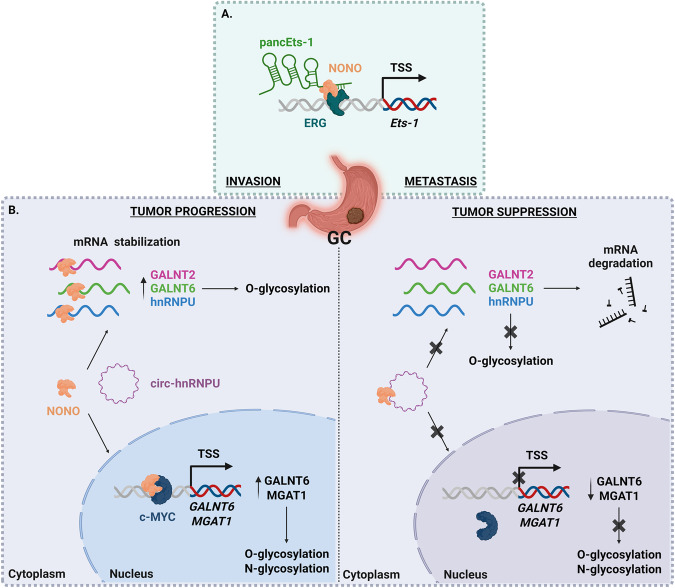
Schematic representation of three NONO molecular mechanisms deregulated in gastric cancer (GC) following NONO overexpression. Simplified pictures of altered mechanisms are shown in (**A**–**C**), see text for detailed description. TSS transcription start site.

Gastric cancer (GC) is one of the leading causes of cancer-related death around the world, and the outcome of patients at advanced disease stages still remains poor, mainly due to tumor recurrence, invasion, and metastasis [54]. NONO expression level is increased in GC tissues compared with normal and precancerous gastric mucosa in association with the poor prognosis of stomach cancer [45]. It has been demonstrated that NONO regulates the expression of V-ets erythroblastosis virus E26 oncogene homolog 1 (Ets-1), one member of the E26 transformation-specific (Ets) family of transcription factors. Ets-1 levels are elevated in GC, and knockdown of Ets-1 inhibits the invasiveness and metastasis of GC cells [76]. Mechanistically, NONO interacts with *Ets-1* promoter-associated noncoding RNA (pancEts-1), which is a lncRNA upregulated and associated with poor survival of GC [45]. In detail, pancEts-1 directly interacts with NONO to increase its interaction with the Ets-related gene (ERG), resulting in increased transactivation of ERG, transcription of Ets-1, and promotion of growth and aggressiveness of GC cells, overall suggesting a crucial role of the pancEts-1/NONO/ERG/Ets-1 axis in GC progression (Fig. 4A) [45].

Very recently, another study discovered an important interaction of NONO with circ‑hnRNPU, a circular RNA (namely a type of endogenous single-stranded and covalently closed non-coding RNA) that derives from the oncogene *hnRNPU* and exerts tumor suppressive roles in protein glycosylation and progression of GC [77]. In particular, circ‑hnRNPU binds NONO and induces its cytoplasmic retention via physical interaction, impairing the dual NONO activity in regulating c‑Myc transactivation and mRNA stabilization. Indeed, NONO sequestration by circ‑hnRNPU results in the down-regulation of both parental hnRNPU and glycosyltransferases genes via repression of nuclear NONO-mediated c-Myc transactivation, and through the reduction of cytoplasmic NONO-facilitated mRNA stabilization (Fig. 4B) [77]. Based on these notions, the circ-hnRNPU/NONO/c-Myc axis may be a potential therapeutic target for GC.

## Prostate cancer

Prostate cancer (PCa) is one of the most common cancers in men worldwide and a leading cause of cancer mortality [78]. Since the androgen receptor (AR) is required for PCa pathogenesis, androgen deprivation therapy has been the principal treatment for aggressive PCa; however, despite the high initial response rates, these tumors ultimately develop resistance, i.e., castration-resistant PCa (CRPC) [79]. Recent studies have demonstrated that constitutive expression of AR splice variants lacking the ligand binding domain significantly contributes to the development and progression of CRPC [80]. Among these, the most studied AR splice variant, AR-V7 or AR3, activates AR-regulated genes in the absence of ligands and therefore could play a critical role in the development of castration resistance.

**Fig. 5:**
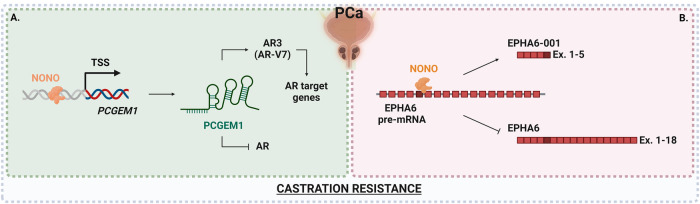
Schematic representation of two NONO molecular mechanisms deregulated in prostate cancer (PCa) following NONO overexpression. Simplified pictures of altered mechanisms are shown in (**A**, **B**), see text for detailed description. TSS transcription start site.

NONO is highly expressed in PCa cells; moreover, the higher expression of NONO correlates with the poor prognosis of patients [43]. Current studies indicate that NONO could affect tumorigenesis and androgen deprivation therapy resistance based on different molecular mechanisms (Fig. 5).

In particular, NONO is a positive regulator of the lncRNA PCGEM1, which contributes to AR3 variant expression and finally fuels castration resistance [33] (Fig. 5A). Furthermore, as described above, NONO is directly involved in AS processes, possibly generating alternative transcript variants that could be crucial in drug resistance. This is the case of ephrin type-A receptor 6 (EPHA6), which encodes a tyrosine kinase receptor that exerts complex activities in cancer and is consistently overexpressed in metastatic PCa cells. In detail, EPHA6 promotes angiogenesis and PCa metastasis and is associated with human PCa progression [81]. Interestingly, overexpression of NONO induces differential EPHA6 splicing generating a truncated transcript. Although further studies are needed to confirm these data in vitro and in vivo, the truncated EPHA6 transcript appears to contribute to castration-resistant PCa growth (Fig. 5B) [34].

Interestingly, a recent paper confirms the relevance of NONO in regulating oncogenic transcriptome in PCa cells by identifying small molecules able to bind NONO thus remodeling the transcriptome of cancer cells, ultimately impairing cell proliferation [82]. Of note, the use of these covalent NONO ligands that stabilize NONO–RNA interactions has very broad implications. Indeed, it is now well established that NONO, SFPQ, and PSPC1 can functionally counterbalance each other in some biological context [1]. In light of this notion, this study highlights the value of selective chemical compounds for studying proteins with functions that may be obscured following genetic disruption due to the compensatory actions of paralogs [82].

## Hepatocellular carcinoma

**Fig. 6:**
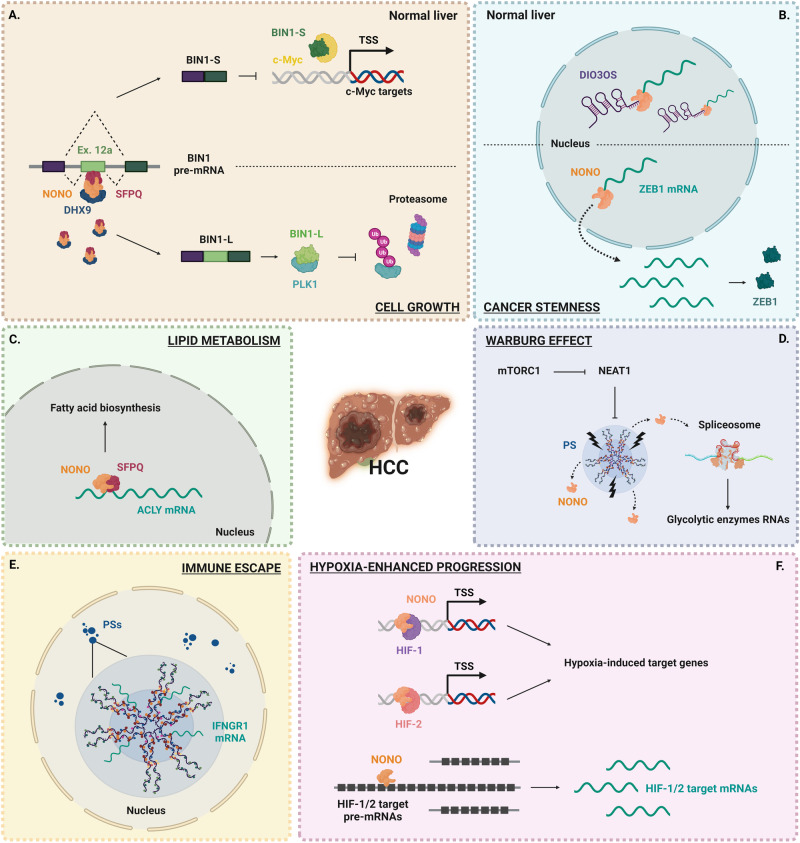
Schematic representation of six NONO molecular mechanisms deregulated in Hepatocellular carcinoma (HCC) following NONO overexpression. Simplified pictures of altered mechanisms are shown in (**A**–**F**), see text for detailed description. PS paraspeckle, TSS transcription start site.

Hepatocellular carcinoma (HCC) is the third leading cause of cancer-related death worldwide, with a dismal survival rate due to a limited understanding of molecular pathogenesis and few available therapeutic options [54]. Recent studies have highlighted NONO as a leading player in HCC tumorigenesis, and high expression of NONO coupled with SFPQ has promising prognostic implications for HCC [83]. Increased NONO expression levels promote tumorigenesis in liver cancer cells based on different molecular mechanisms (Fig. 6).

For instance, NONO functions as an oncogene by regulating the splicing switch of MYC box-dependent interacting protein 1 (BIN1), a process that is dependent on the DExH-box helicase 9 (DHX9)–NONO–SFPQ complex. Under moderate NONO expression, normal liver cells generate BIN1 short isoform (BIN1-S) to restrain cell growth through inhibition of the binding of c-Myc to target gene promoters. In HCC, NONO is highly expressed and modulates the splicing switch of BIN1-S to generate BIN1 long isoform (BIN1-L), which contains exon 12a and plays an oncogenic role through association with polo-like kinase 1 (PLK1) to enhance its protein stability [83], and finally promoting cell cycle progression (Fig. 6A).

Another study reported that NONO can affect HCC stemness based on its interaction with DIO3OS, a lncRNA conserved across various species and generally downregulated in multiple cancers. This study demonstrated that low DIO3OS expression levels in HCC allow NONO to mediate the Zinc finger E-box-binding homeobox 1 (ZEB1) mRNA nuclear export, thereby enhancing liver tumorigenesis, and particularly HCC cells stemness [84]. NONO involvement in nuclear export could be considered a novel biological function of NONO in mRNA biology. Furthermore, although the study was focused on DIO3OS, we cannot rule out that, beyond low levels of DIO3OS expression, also high levels of NONO expression may regulate the nuclear export of ZEB1 mRNA in HCC (Fig. 6B).

NONO supports HCC progression by affecting cancer cell metabolism. In complex with SFPQ, NONO directly interacts with ATP-citrate lyase (ACLY) mRNA and enhances its nuclear stability, finally inducing fatty acids biosynthesis (Fig. 6C) [85]. Furthermore, NONO is involved in the reprogramming of glucose metabolism from respiration (oxidative phosphorylation) to aerobic glycolysis, a phenomenon known as the ‘Warburg Effect’. In particular, upon oncogenic activation, the mechanistic target of rapamycin complex 1 (mTORC1) suppresses the lncRNA NEAT1 expression and PS biogenesis, releasing NONO, which in turn binds to spliceosome, stimulating mRNA splicing and expression of key glycolytic enzymes. This series of actions leads to enhanced glucose transport, aerobic glycolytic flux, lactate production, and HCC growth both in vitro and in vivo [86] (Fig. 6D).

In the context of the PS organelle, NONO can sequester the interferon-gamma receptor 1 (IFNGR1) mRNA in HCC cancer cells, promoting tumor cells escape from immunosurveillance by T-cells [87] (Fig. 6E).

Finally, NONO plays a crucial role in HCC cells by driving angiogenesis and glycolysis, two well-known cancer phenotypes mediated by hypoxia. Indeed, NONO interacts with and stabilizes both hypoxia-inducible factors 1 and 2 (HIF-1 and HIF-2) complexes, thus activating the transcription of hypoxia-induced genes. Besides, NONO binds pre-mRNA and subsequent mRNAs of these genes to facilitate both splicing and mRNA stability, promoting the hypoxia-enhanced progression in HCC [88] (Fig. 6F).

## Melanoma

Melanoma is a malignant tumor affecting cutaneous melanocytes. Exposure to ultraviolet (UV) radiation, namely UVA (315–400 nm) and UVB (280–315 nm), is a major risk factor for melanoma development, as it can cause direct DNA damage. NONO contributes to rapid and accurate repair of DNA double-strand breaks in human cells [27]; furthermore, NONO silencing negatively impacts the UVC (100–280 nm)-induced DNA damage response in melanoma cells [89]. Based on these considerations, NONO could be considered an efficient target of radio-sensitizing agents.

NONO is highly expressed in melanoma cells and its transcription is positively regulated by the melanoma inhibitory activity (MIA), a small soluble secreted protein strongly expressed in malignant melanoma cells but absent in normal human melanocytes, which is functionally important both in early tumor formation and in melanoma progression [36, 90, 91]. In detail, MIA supports melanoma development through activation of the transcription factor Y-box binding protein 1 (YBX1), which in turn enhances NONO transcription [91]. Recently, it has been reported that NONO promotes the transcription of oncogenic genes in melanoma, among which the tumorigenic protease cathepsin-Z and the anti-apoptotic gelsolin [92].

**Fig. 7:**
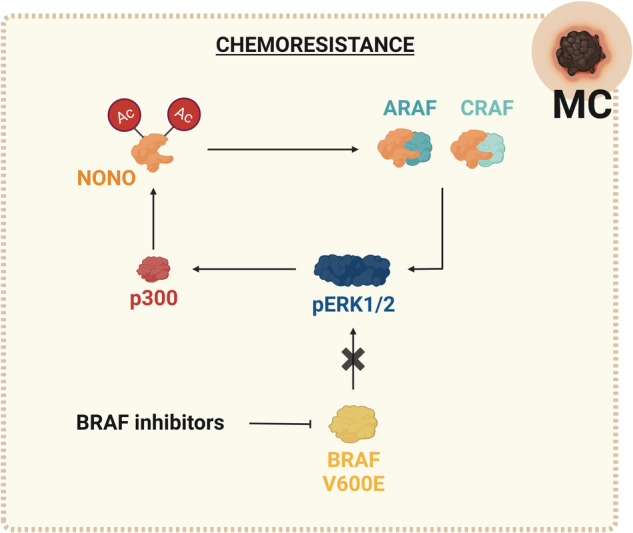
Schematic representation of NONO molecular mechanisms altered by NONO deregulation in melanoma (MC). See the text for a detailed description.

A peculiar role of NONO has been described in nearly half of melanoma patients carrying the mutation V600E in the *BRAF* kinase; for this subset of patients, BRAF inhibitors show a significant antitumor response, but the common emergence of acquired resistance remains a challenge. Indeed, based on mechanisms still unclear, melanoma cells show a dysregulated expression of the RAF isoforms ARAF and CRAF, which reactivate pERK1/2 playing a crucial role in the acquisition of resistance. Notably, NONO interacts with and stabilizes both ARAF and CRAF in melanoma cells; moreover, NONO is acetylated by p300 acetyltransferase, which stabilizes NONO antagonizing its ubiquitination/degradation. Interestingly, the NONO/ARAF/CRAF-mediated pERK1/2 activation leads to an increased expression of p300, which leads to increased acetylation and stability of NONO, thus sustaining a positive regulatory feedback loop in drug-resistant melanoma cells. Ultimately, the upregulation of both p300 and NONO favors the rebound of pERK1/2 and the development of subsequent resistance of melanoma cells to BRAF inhibitors. Hence, targeting the positive feedback loop of p300-NONO-CRAF/ARAF-pERK1/2 may be an effective strategy to overcome the resistance of BRAF inhibitors for melanoma patients [93] (Fig. 7).

## Multiple myeloma

Multiple myeloma (MM) is a hematologic malignancy that is still incurable despite the remarkable improvements in treatment and patient care. MM is characterized by a highly heterogeneous genetic background with both structural chromosomal alterations and specific gene mutations, which are associated with the clinical heterogeneity of the disease, and whose identification is potentially relevant for improving tailored therapeutic approaches [94, 95].

**Fig. 8:**
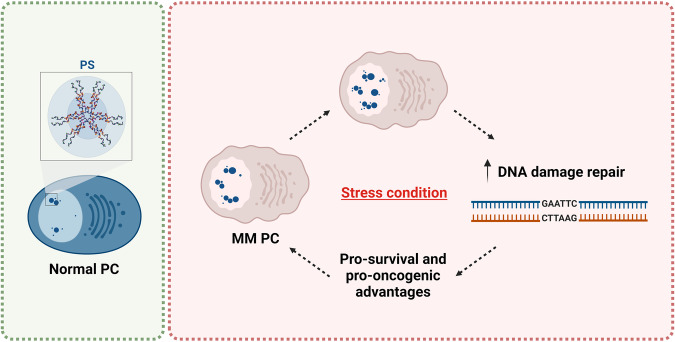
Schematic representation of NONO molecular mechanisms altered by NONO deregulation in multiple myeloma (MM). PS paraspeckle. See the text for a detailed description.

Recently, NONO has been reported to be highly expressed in MM plasma cells compared to the normal counterpart, and its expression levels are significant prognostic markers of clinical outcomes [35]. Its crucial role in MM was initially identified in the context of PSs. In particular, MM plasma cells increase PSs number and dimension to counteract different stimuli including stressful conditions. The increase of PSs, and therefore of NONO, is crucial to sustain the growth and the survival potential of MM cells in both serum starvation and hypoxia, which are typical stressful conditions for tumor cells in vivo and are often associated with more aggressive tumor stages and mechanisms of chemoresistance. In addition, a functional contribution of PSs in the DNA damage response pathway could be hypothesized. In this scenario, PSs could be considered a generalized rescue mechanism for MM plasma cells under stressful conditions suggesting that PS targeting could be a promising novel strategy for innovative anti-MM therapies effective for all subsets of MM patients [96] (Fig. 8).

However, besides its essential role within PSs, a fraction of NONO is localized outside these nuclear bodies, suggesting that NONO could play important roles independently from them. In line with this notion, NONO is found involved in the metabolic reprogramming of glucose metabolism from respiration to aerobic glycolysis [35], namely the Warburg Effect already described in HCC, that supports rapid cancer cell growth, survival, and invasion [97].

## Conclusion

An increasing amount of data is emerging on the molecular mechanisms through which NONO promotes carcinogenesis. However, the landscape of NONO activities and interactions is extremely broad and complex enough to conceal several caveats that must be taken into account while investigating its function.

First of all, NONO belongs to the DBHS family of dynamic proteins, mediating a wide range of protein–nucleic acid and protein–protein interactions, on the whole acting as a versatile molecular scaffold. NONO, SFPQ, and PSPC1 essentially act as dimers and the different protein combinations likely have different functions. As a consequence, DBHS proteins can functionally counterbalance each other in some biological conditions, but not in others [1]. Hence, experimental planning should ponder the compensatory actions of paralogs.

The scenario is even more complex, as the cellular pool of DBHS proteins is constantly regulated in terms of protein abundance and localization, thus allowing their dynamic and context-dependent functions. For example, to coordinate circadian rhythm and cell cycle, NONO protein expression is not rhythmic whereas SFPQ protein levels appear to oscillate with the circadian cycle [29]. As a result, both the redundant and protein-specific functions of the nucleoplasmic pool of different DBHS proteins are affected by the circadian cycle maintenance, indicating that the choice of experimental timing should be carefully considered.

Another important point to take into account is the subcellular localization of the DBHS proteins. Besides their subnuclear positioning within PSs, or in the nucleoplasmic pool, DBHS proteins can also reside outside the nucleus [77, 98, 99]. Given their abundance and dynamic nature, the cytoplasmic role of DBHS proteins may have been underestimated to date.

Equally undervalued could also be the presence of a heterogeneous population of NONO isoforms. Indeed, at least two main protein variants have been described, which may not be immunodetected by common commercial monoclonal antibodies as it has been found in BC [38].

Based on these considerations, some past literature concerning NONO, where it is annotated as an individual functional unit, may need to be reinterpreted. However, these studies clearly indicate that NONO plays a central role in the majority of tumor types by affecting many key pathways involved in proliferation, metastasis, and chemoresistance based on multiple molecular mechanisms. Overall, these results strongly suggest that a better understanding of the context of NONO’s functions in cells and tumorigenesis will make it therapeutically invaluable; furthermore, they pave the way for future studies that cannot ignore the dynamic expression and interplay between DBHS protein paralogs, especially given functional overlap and redundancy.
